# I-gel Plus acts as a superior conduit for fiberoptic intubation than standard i-gel

**DOI:** 10.1038/s41598-023-45631-0

**Published:** 2023-10-26

**Authors:** Tomohiro Chaki, Shunsuke Tachibana, Sho Kumita, Satoshi Sato, Tomoki Hirahata, Yuta Ikeshima, Yuki Ohsaki, Michiaki Yamakage

**Affiliations:** 1 Department of Anesthesiology, Sapporo Medical University School of Medicine, Sapporo, Japan; 2 Department of Anatomy (I), Sapporo Medical University School of Medicine, Sapporo, Japan

**Keywords:** Preclinical research, Respiratory tract diseases

## Abstract

The supraglottic airway (SGA) is widely used. I-gel Plus is a next-generation i-gel with some improvements, including facilitation of fiberoptic tracheal intubation (FOI). To compare the performance of i-gel Plus and standard i-gel as conduits for FOI, a Thiel-embalmed cadaveric study was conducted. Twenty-two anesthesiologists were enrolled as operators in Experiment 1. The i-gel Plus and standard i-gel were inserted into one cadaver, and the FOI was performed through each SGA. The primary outcome was time required for FOI. The secondary outcomes were the number of attempts and visual analog scale (VAS) score for difficulty in FOI. Moreover, fiberoptic views of the vocal cords in each SGA were assessed by an attending anesthesiologist using nine cadavers in Experiment 2. The percentage of glottic opening (POGO) score without fiberscope tip upward flexion and upward angle of the fiberscope tip to obtain a 100% POGO score were evaluated as secondary outcomes. The time for FOI through i-gel Plus was significantly shorter than that through standard i-gel (median (IQR), i-gel Plus: 30.3 (25.4–39.0) s, vs standard i-gel: 54.7 (29.6–135.0) s; median of differences, 24.4 s; adjusted 95% confidence interval, 3.0–105.7; adjusted *P* = 0.040). Although the number of attempts for successful FOI was not significantly different, the VAS score for difficulty in the i-gel Plus group was significantly lower (easier) than that in the standard i-gel group. Moreover, i-gel Plus required a significantly smaller upward angle of the fiberscope tip to obtain a 100% POGO score. FOI can be performed more easily using i-gel Plus than using standard i-gel because of the improved fiberoptic visibility of vocal cords.

## Introduction

The effectiveness of supraglottic airway (SGA) devices has been elucidated by numerous studies, including clinical and experimental studies, and the use of SGA has expanded beyond anesthesiology and emergence medicine^1–3^. The first-generation SGA devices do not permit gastric tube placement, although it can be performed using second-generation SGA devices. SGAs can be used for rescue airway management in difficult airways^4,5^ and are useful as conduits for tracheal intubation in a blind manner or with a fiberscope^6–8^.

One of the second-generation SGAs is i-gel (Intersurgical Ltd., Wokingham, UK). It has a characteristic non-inflatable cuff that can change its shape according to body temperature and provide a better fit for individual patients after insertion^9,10^. Additionally, the standard i-gel has a high rate of successful insertion on the first attempt and a low incidence of bloodstains caused by airway injury^11,12^. Therefore, it has been widely used in various situations, including laparoscopic surgery and prone positioning^13,14^.

I-gel Plus (Intersurgical Ltd.) is an improved version of i-gel. The improved features of i-gel Plus include a supplementary oxygen port, larger gastric channel, airway tube optimized for use as a conduit for tracheal intubation, and longer non-inflatable cuff tip for improving oropharyngeal leak pressure. With these improvements, the clinical performance of the i-gel Plus may exceed that of the standard i-gel. A clinical cohort study evaluating the performance of i-gel Plus is currently being conducted in Europe^15^. However, the results of this study have not yet been published. Moreover, this cohort study did not include a comparative group. Thus, the performance of i-gel Plus compared to that of other SGA devices remains unclear.

We hypothesized that i-gel Plus would be superior as a conduit for fiberoptic tracheal intubation (FOI) compared to the standard i-gel. However, the clinical use of the i-gel Plus has not yet been approved in Japan. The Thiel-embalming method has the benefit of maintaining the mobility of joints and soft tissue properties close to those of the living body, unlike formalin fixation^16^. A Thiel-embalmed cadaver is considered suitable for airway management training^17^. Therefore, prior to the clinical use of i-gel Plus, we conducted a Thiel-embalmed cadaveric study to investigate the performance of the new i-gel Plus as a conduit for FOI compared with the conventional standard i-gel.

## Materials and methods

This study was conducted in accordance with the Sapporo Medical University guideline for the Use of Donated Bodies. This cadaveric study was approved by the Ethics Committee of Sapporo Medical University School of Medicine (approval code: 4-1-88) and used Thiel-embalmed cadavers that were donated to the body donation program (Shiragiku-kai) run by Sapporo Medical University for medical scientific research. This cadaveric study was conducted from February 19 to 28, 2023, when permission to use cadavers was obtained from the Ethics Committee. I-gel Plus and i-gel were purchased from Intersurgical Ltd. with research funds from the Department of Anesthesiology, Sapporo Medical University School of Medicine. Written informed consent was obtained from all participating anesthesiologists and the next of kin of donors prior to this study. Informed consent obtained from the next of kin of donors included both standard autopsies (for anatomy training) and clinical autopsies (for clinical research). No organs, tissues, or cadavers were procured from prisoners in this study.

### Experiment 1: performance as a conduit for FOI

**Figure 1:**
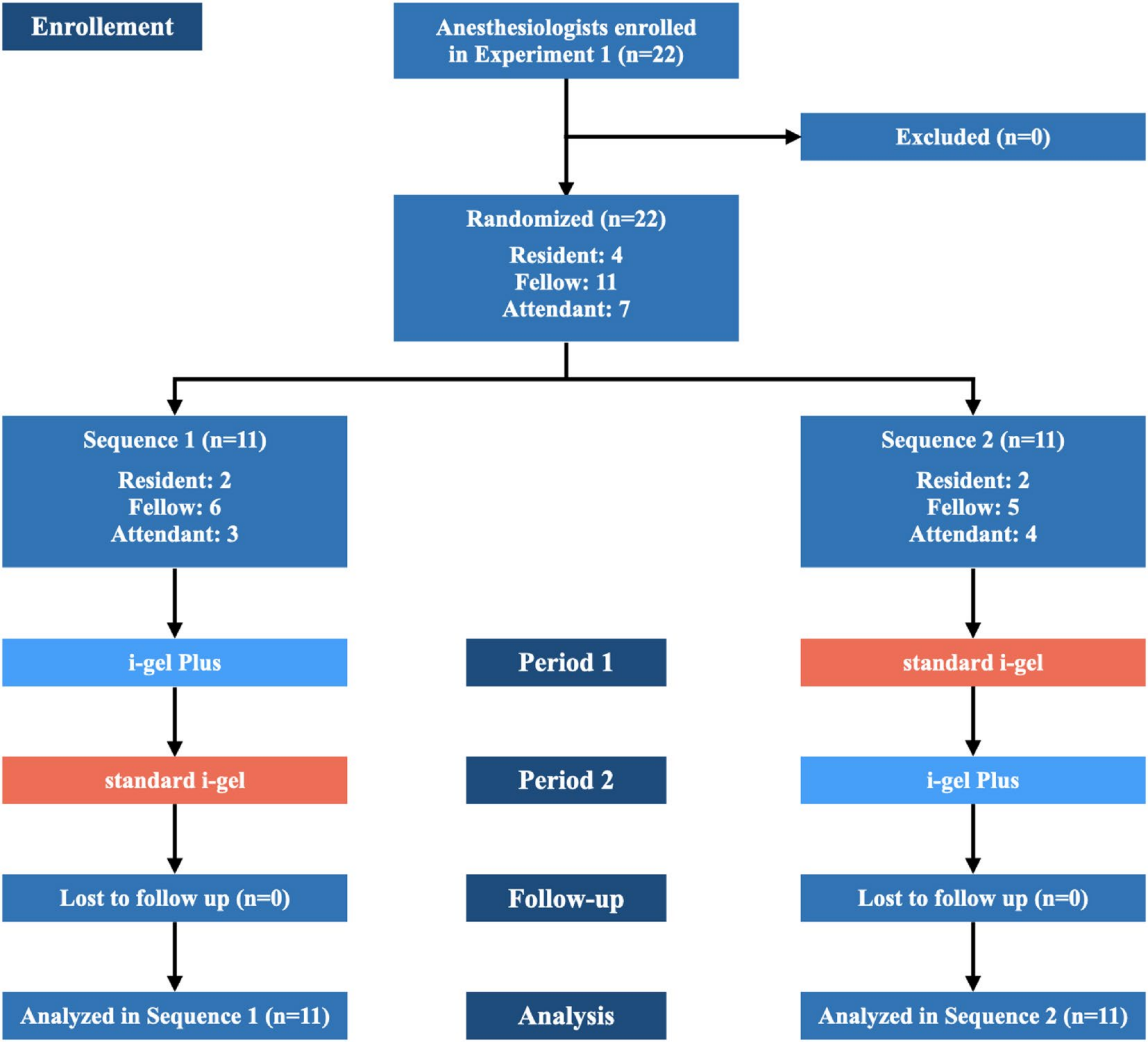
Flow diagram of randomization for Sequences 1 and 2 in Experiment 1.

In Experiment 1, a female cadaver with no damage to her whole body was used to evaluate the performance of i-gel Plus and standard i-gel. A crossover design was used in Experiment 1, and the participating anesthesiologists were randomly divided into Sequence 1 (i-gel Plus and standard i-gel were evaluated in that order) and Sequence 2 (standard i-gel and i-gel Plus were evaluated in that order) (Fig. 1). To avoid bias at the anesthesiologist level, randomization was performed using envelope random methods at the resident, fellow, and attendant levels of anesthesiologists.

Each participating anesthesiologist inserted a size 3 i-gel Plus and a size 3 standard i-gel in a randomized order and evaluated the outcomes. The cadaver was placed in a semi-sniffing position and SGA insertion was performed. Successful SGA insertion was defined as confirmation of chest elevation by positive pressure ventilation using a bag valve mask system (SPUR II; Ambu, Ballerup, Denmark). After successful insertion, tracheal intubation was performed through the airway tube of the SGA device using a fiberscope (Ambu aScope Broncho regular, Ambu). A tracheal tube with an internal diameter of 6.0 mm (TaperGuard, Covidien, Dublin, Ireland) was used for intubation. The number of attempts and time required for successful intubation and the visual analog scale (VAS) score for difficulty in FOI were evaluated. Tracheal intubation was considered successful when chest elevation was confirmed by positive-pressure ventilation. The time required for tracheal intubation was determined as the time from fiberscope insertion into the airway lumen of the SGA to confirmation of chest elevation by positive-pressure ventilation. After successful tracheal intubation, the VAS score for difficulty in FOI was evaluated (0, easiest; 100, most difficult). After evaluating the first SGA device, the outcomes of the second SGA device were evaluated by the same anesthesiologist using the same procedure, according to the randomized sequence.

### Experiment 2: fiberoptic view of vocal cords

In Experiment 2, one attending anesthesiologist performed all SGA insertions and evaluated the fiberoptic view of the vocal cords. Both the i-gel Plus and standard i-gel were inserted into each of the cadavers with intact airways. The fiberoptic percentage of the glottic opening (POGO) score without upward flexion of the tip of the fiberscope and the upward flexion angle of the fiberscope tip needed to secure a 100% POGO score were evaluated through i-gel Plus and standard i-gel, respectively. Sizes 3 and 4 of the SGA devices were inserted into the female and male cadavers, respectively. After insertion, a fiberscope was passed through the airway lumen of the SGA device to a position 1 cm proximal to the airway lumen tip, and a fiberoptic view of the vocal cords score was observed. The score was recorded as 4, only vocal cords visible; 3, vocal cords and posterior side of epiglottis visible; 2, vocal cords and anterior side of epiglottis visible; and 1, vocal cords not seen^18,19^. Fiberoptic views of vocal cords with scores of 3 or 4 were considered successful SGA device insertions. After confirming successful placement of the SGA device, the POGO score without any flexion of the fiberscope tip was evaluated. Thereafter, the tip of the fiberscope was flexed upward to ensure a 100% POGO score, and the upward flexion angle of the fiberscope tip was measured using a digital protractor after removal of the fiberscope from the SGA device.

The primary outcome of this study was the time required for the FOI. The secondary outcomes were the number of attempts at successful intubation and the VAS score for difficulty in FOI. Moreover, the fiberoptic POGO score without upward flexion of the fiberscope tip and the upward flexion angle of the fiberscope tip needed to secure a 100% POGO score were evaluated as secondary outcomes.

### Statistical analysis

Statistical analyses were performed using GraphPad Prism9 (GraphPad Software, Boston, MA). Normality was confirmed using the Shapiro–Wilk test. If the data had a normal distribution, they were presented as mean (standard deviation). Meanwhile, if the data had a non-normal distribution, they were presented as median (interquartile range). The time and number of attempts required for successful FOI and the VAS for difficulty in FOI were analyzed using two-way analysis of variance (ANOVA). If two-way ANOVA revealed a significant interaction between Sequence and Period, Mann–Whitney *U* test with Bonferroni correction was used for the analysis between i-gel Plus and standard i-gel in Periods 1 and 2, respectively. The Mann–Whitney *U* test was used for carryover effect analysis. Moreover, the fiberoptic POGO score and upward flexion angle of the fiberscope tip for the 100% POGO score were analyzed using the Wilcoxon signed-rank test. The proportion of anesthesiologists’ level was analyzed using chi-square test. A two-tailed *P* and adjusted *P* value < 0.05 was considered statistically significant.

According to a previous study, a total of 20 participants were needed based on two-tailed α = 0.05, 1 − β = 0.9, and the clinically relevant mean difference in SGA-guided intubation time of 20 s, with a standard deviation of 25 s^20^. Considering dropouts or exclusions, we added 10% of the participants and finally determined that 22 anesthesiologists were necessary.

### Ethics approval

This cadaveric study was approved by the Ethics Committee of Sapporo Medical University School of Medicine (approval code: 4-1-88).

## Results

Cadaveric characteristics are listed in Table 1. One female cadaver (cadaver number 1 in Table 1; age, 70 years; height, 155 cm; weight, 49.0 kg) was used in Experiment 1. In Experiment 2, six female and three male cadavers were used (Table 1). The mean age was 84.3 (9.4) years. The mean height and weight were 151.8 (9.7) cm and 46.3 (11.7) kg, respectively. In Experiment 1, 22 anesthesiologists were recruited to participate in the study. Two residents, six fellows, and three attendants were assigned Sequence 1. Two residents, five fellows, and four attendants were allocated to Sequence 2 (Fig. 1). The proportion of anesthesiologists was comparable at each level (*P* = 0.953). Because there was no data exclusion, 22 pairwise data points were finally analyzed for each outcome of Experiment 1.

**Table 1:** Characteristics of cadavers.

Number	Sex	Age (years)	Height (cm)	Weight (cm)	BMI (kg/m^2^)
1	F	70	155	49.0	20.4
2	M	50	153	65.0	27.8
3	M	75	163	51.3	19.3
4	F	77	158	44.0	17.6
5	M	91	162	50.6	19.3
6	F	84	145	41.4	19.7
7	F	96	140	28.9	14.7
8	F	91	155	56.4	23.5
9	F	95	135	30.0	16.5
Summary	F/M = 3/6	84.3 (9.4)	151.8 (9.7)	46.3 (11.7)	19.9 (3.9)

*BMI* body mass index, *F* female, *M* male.

Data are presented as number or mean (standard deviation).

### Time required for FOI

There was no carryover effect in the analysis of time for FOI according to the Mann–Whitney *U* test (Supplementary Table 1). However, two-way ANOVA revealed a significant interaction in the time for intubation (*P* = 0.011), indicating a possible carryover effect (Supplementary Fig. 1a). In Period 1, the time required for the FOI was significantly shorter with i-gel Plus than with the standard i-gel (Table 2). On the other hand, there was no significant difference in time for FOI between i-gel Plus and standard i-gel in Period 2 (Table 2).

**Table 2:** Fiberoptic tracheal intubation using i-gel Plus and standard i-gel.

	i-gel Plus (n = 11)	Standard i-gel (n = 11)	Median of differences	95% CI	Adjusted *P* value
Time (s)					
Period 1	30.3 (25.4–39.0)	54.7 (29.6–135.0)	24.4	3.0 to 105.7	0.040
Period 2	30.7 (17.1–34.1)	41.9 (26.8–53.4)	11.2	− 5.7 to 28.6	0.199
VAS (mm)					
Period 1	33.0 (17.0–46.0)	67.0 (40.0–88.0)	34.0	8.0 to 58.0	0.007
Period 2	31.0 (24.0–37.0)	46.0 (33.0–50.0)	15.0	− 4 to 25.0	0.106
Attempts (N)					
Period 1	1 (1–1)	1 (1–2)	0	0 to 1	0.070
Period 2	1 (1–1)	1 (1–2)	0	0 to 1	0.677

*CI* confidence interval, *VAS* visual analog scale.

Data are presented as absolute number or median (interquartile range).

Mann–Whitney *U* test with Bonferroni correction was used for the statistical analysis.

Adjusted *P* value < 0.05 was considered as statistically significant.

### VAS for difficulty of FOI

There was no carryover effect in the analysis of VAS for difficulty of FOI according to the analysis Mann–Whitney *U* test (Supplementary Table 1). However, two-way ANOVA clarified that there was a significant interaction in the VAS for intubation (*P* < 0.001), which indicated that there was a possible carryover effect (Supplementary Fig. 1b). Although there was no significant difference in the VAS for difficulty of fiberoptic intubation between i-gel Plus and standard i-gel in Period 2, the VAS for difficulty of FOI was significantly lower (easier) with i-gel Plus than with standard i-gel in Period 1 (Table 2).

### Attempt number of FOI

There was no carryover effect in the analysis of the number of attempts for FOI (Supplementary Table 1). There was no significant difference between the i-gel Plus and standard i-gel in Periods 1 and 2, respectively.

### Fiberoptic view of vocal cords

**Figure 2:**
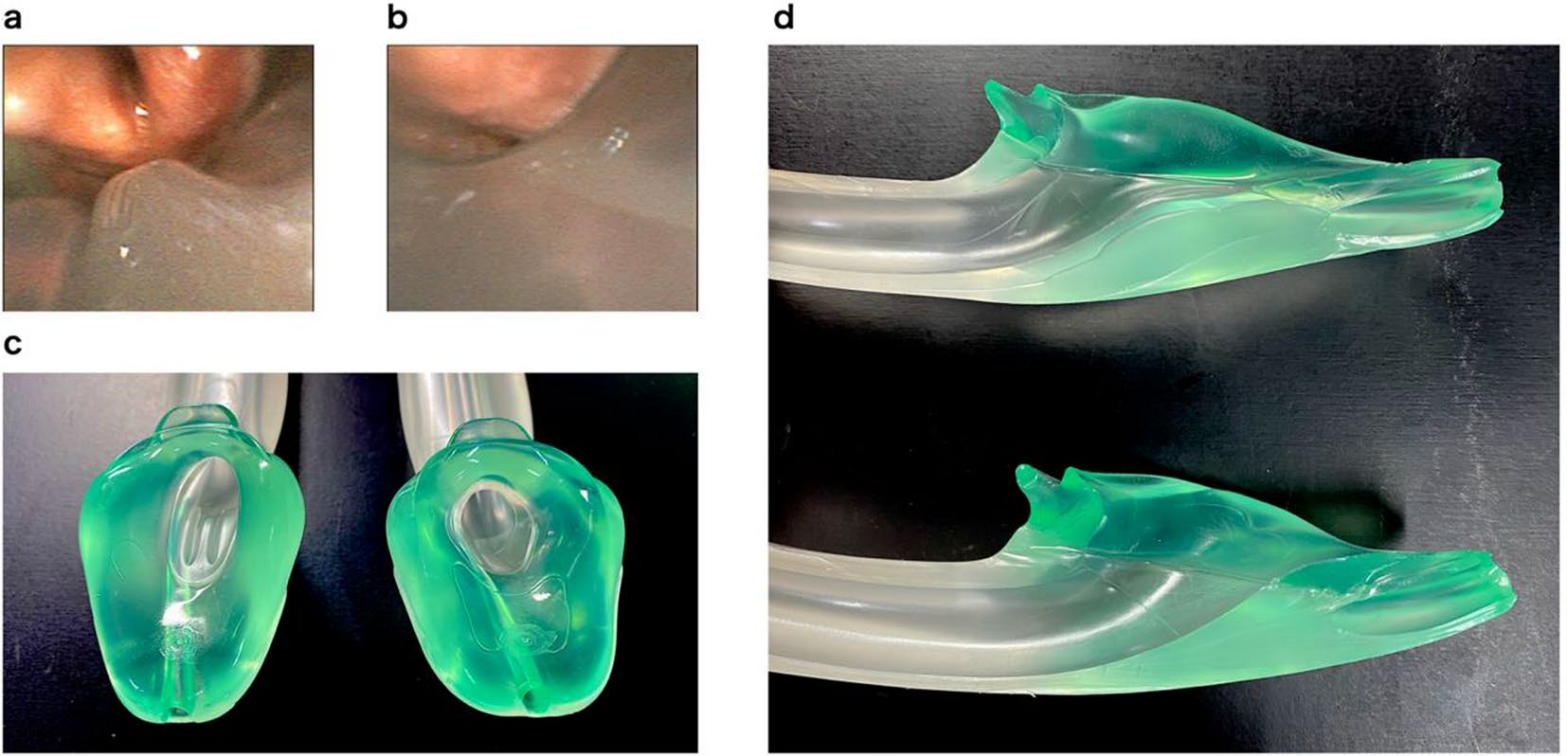
Fiberoptic view of the vocal cords and appearance of i-gel Plus and standard i-gel. (**a**) Representative images of the fiberoptic view of the vocal cords without upward flexion of the fiberscope tip in i-gel Plus and (**b**) standard i-gel. The lower part of the vocal cords was visible without upward flexion of the fiberscope tip in i-gel Plus. Meanwhile, only arytenoid cartilages, not vocal cords, were visible in standard i-gel. The fiberoptic views of the vocal cords were obtained from the same female cadaver. (**c**) Airway tube orifice of i-gel Plus and standard i-gel. The left and right devices were the i-gel Plus and i-gel, respectively. (**d**) Midline cross-section of the airway tube of i-gel Plus and standard i-gel. The upper and lower devices were the i-gel Plus and i-gel, respectively. I-gel Plus has a ramp in the airway tube orifice to optimize the direction of the fiberscope and tracheal tube for intubation.

In Experiment 2, a total of nine pairwise data were analyzed for each outcome, because there was no data exclusion. Although no significant difference in POGO scores without fiberscope tip flexion was observed, i-gel Plus required a significantly smaller upward angle of the fiberscope tip to obtain a 100% POGO score than the standard i-gel (Table 3). Representative fiberoptic views of the vocal cords of the i-gel Plus and standard i-gel without upward flexion of fiberscope tip are presented in Fig. 2a,b, respectively. Supplementary Video 1 and Supplementary Video 2 are representative movies of the fiberoptic view of the vocal cords of i-gel Plus and standard i-gel, respectively.

**Table 3:** Fiberoptic view of vocal cords using i-gel Plus and standard i-gel.

	i-gel Plus (n = 9)	Standard i-gel (n = 9)	Median of differences	95% CI	*P* value
POGO score without fiberscope tip flexion (%)	60.0 (12.5–70.0)	0 (0–35.0)	15.0	− 5.0 to 55.0	0.051
Upward angle of fiberscope tip to obtain a 100% POGO score (degree)	53.0 (45.0–67.5)	73.0 (69.0–77.5)	20.0	12.0 to 27.0	0.004

*CI* confidence interval, *POGO* percentage of glottic opening.

Data are presented as median (interquartile range).

Wilcoxon signed rank test was used for the statistical analysis.

## Discussion

In this cadaveric study, we compared the performance of i-gel Plus and standard i-gel as conduits for FOI. The time needed for FOI was significantly shorter with the i-gel Plus than with standard i-gel. Moreover, the VAS score for difficulty in FOI was significantly smaller with i-gel Plus than with standard i-gel. The upward flexion of the fiberscope to ensure a 100% POGO score in i-gel Plus was smaller than that in standard i-gel. The improved visibility of the vocal cords might make FOI easier with i-gel Plus.

I-gel Plus is a next-generation i-gel with improved clinical features. One of the improvements is that the airway tube was optimized for FOI (Fig. 2c,d). Some types of SGA devices, including the standard i-gel, can be used as conduits for fiberscope-guided tracheal intubation, and this technique is useful for difficult intubations^6–8^. Some studies comparing the efficacy of SGA devices as conduits for FOI have been conducted, although no significant difference in the effectiveness for FOI has been reported between standard i-gel and other SGA devices^21–23^. In contrast, the FOI was easier with i-gel Plus than with standard i-gel, according to our results. Although there is no difference between i-gel Plus and standard i-gel in the lumen size of the airway tube and the maximum diameter of the intubation tube through which the airway tube can be passed, the improvement in the orifice of the i-gel Plus airway tube may allow easier FOI. The mechanism for the ease of FOI is to improve the visibility of the vocal cords using a fiberscope, which was confirmed by evaluating the upward flexion angle of the fiberscope tip to ensure a 100% POGO score between i-gel Plus and standard i-gel in this study. I-gel Plus has a ramp in the airway tube orifice to optimize the direction of the fiberscope and tracheal tube for intubation (Fig. 2c,d). It is thought that the anesthesiologists could observe the vocal cords using a fiberscope more quickly and perform tracheal intubation earlier in i-gel Plus (Fig. 2d).

Although the time required for tracheal intubation and the VAS for difficulty in FOI were significantly different in Period 1, they were not significantly different in Period 2. In Period 2, it is possible that the anesthesiologists were more familiar with the FOI procedure through SGA than in Period 1, and the difference in performance for the FOI could not be detected due to the small sample size. However, in clinical settings, the success of tracheal intubation in the first attempt is crucial because multiple attempts at tracheal intubation increase the risk of severe respiratory complications^24,25^. Therefore, it is thought that the FOI was easier in Period 1, which was clinically important for preventing airway complications.

This study has several limitations. First, the sample size is small. Particularly, to clarify the difference in ease of insertion, clinical trials with a larger sample size are mandatory. Second, operators and investigators were not blinded to the SGA device. Although blinding was impossible because of the study protocol, the non-blinding procedure might have caused observer bias, especially in VAS evaluations. Moreover, only one anesthesiologist evaluated the outcomes of Experiment 2. This may have caused an observer bias. Third, this was a cadaveric study and not a clinical study, although we used a Thiel-embalmed cadaver that can maintain tissue flexibility similar to that of a living body compared to a conventional formalin-embalmed cadaver. Our results may not be applicable in clinical settings. Additionally, the cadavers were obtained from an adult Asian population. The upper airway anatomy of Asians and adults may be significantly different from that of other races and children^26–28^. Therefore, our results may not be applicable to other populations or to children. Fourth, the success of SGA insertion was defined by the confirmation of chest elevation by positive pressure ventilation in Experiment 1. In clinical settings, the success of SGA insertion is confirmed by the waveform of capnography; however, this is impossible in cadaveric studies. In Experiment 1, SGA malposition might have occurred, and the results might have been influenced by the possible inappropriateness of SGA placement. Fifth, only one cadaver could be used in Experiment 1 because the remaining eight cadavers had injuries to parts other than the respiratory tract due to surgical training. Therefore, the results of Experiment 1 might have been different in other cadavers with different airway anatomies. Finally, airway evaluations, including dental status, thyromental distance, and Cormack–Lehane grade, were not performed in this cadaveric study. The difficulty of SGA insertion and tracheal intubation in the cadavers remains unclear. Further clinical studies are required because the results of this study may not be applicable in clinical situations, especially in patients with risk factors for difficult SGA insertion and tracheal intubation.

## Conclusions

We compared the performance of i-gel Plus and standard i-gel as conduits for FOI in Thiel-embalmed cadavers. The time for successful FOI using i-gel Plus was shorter than that using standard i-gel. The improved visibility of the vocal cords in the i-gel Plus might facilitate FOI. A large randomized controlled study is necessary to clarify the clinical performance of i-gel Plus as a conduit for FOI.

### Supplementary Information

Supplementary Legends.

Supplementary Table 1.

Supplementary Video 1.

Supplementary Video 2.

Supplementary Figure 1.

## Data Availability

All data are available upon request from the corresponding author.
