# Supplementary material for: I-gel Plus acts as a superior conduit for fiberoptic intubation than standard i-gel
Source: Sci Rep. 2023 Oct 26;13:18381. doi: 10.1038/s41598-023-45631-0 (PMC10603072; doi:10.1038/s41598-023-45631-0)
Supplement: Supplementary file 1 — Supplementary Legends. [file 41598_2023_45631_MOESM1_ESM.docx]

Legend for supplementary videos

Supplementary video 1. Fiberoptic view of vocal cords in i-gel Plus. In the i-gel Plus, the lower part of the vocal cords was visible without upward flexion of the fiberscope tip. Only a small amount of upward flexion of the fiberscope tip allowed observation of the entire vocal cords.

Supplementary video 2. Fiberoptic view of vocal cords in standard i-gel. Without upward flexion of the fiberscope tip, the vocal cords could not be observed, only the arytenoid cartilages. To observe the entire vocal cords, a large amount of upward flexion of the fiberscope tip is necessary.
