# Supplementary material for: I-gel Plus acts as a superior conduit for fiberoptic intubation than standard i-gel
Source: Sci Rep. 2023 Oct 26;13:18381. doi: 10.1038/s41598-023-45631-0 (PMC10603072; doi:10.1038/s41598-023-45631-0)
Supplement: Supplementary file 2 — Supplementary Table 1. [file 41598_2023_45631_MOESM2_ESM.docx]

Supplementary Table 1. Statistical analysis for carryover effect in time, attempt number and visual analog scale for fiberoptic tracheal intubation.

| Outcomes | Sequence 1 | Sequence 2 | *P* value |
| --- | --- | --- | --- |
| Intubation |  | | |
| Time (sec) | 75.3 (57.7–90.3) | 84.3 (45.2–165.7) | 0.449 |
| Attempts (N) | 2 (2–3) | 2 (2–4) | 0.574 |
| VAS (mm) | 116 (108–152) | 103 (70–133) | 0.117 |

Data are presented as median (interquartile range). Mann-Whitney U test was used for the statistical analysis. In Sequence 1, anesthesiologists evaluated i-gel Plus and standard i-gel in that order. On the other hand, in Sequence 2, anesthesiologists evaluated standard i-gel and i-gel Plus in that order. SGA, supraglottic airway device; VAS, visual analog scale.
