# Supplementary material for: I-gel Plus acts as a superior conduit for fiberoptic intubation than standard i-gel
Source: Sci Rep. 2023 Oct 26;13:18381. doi: 10.1038/s41598-023-45631-0 (PMC10603072; doi:10.1038/s41598-023-45631-0)
Supplement: Supplementary file 5 — Supplementary Figure 1. [file 41598_2023_45631_MOESM5_ESM.docx]

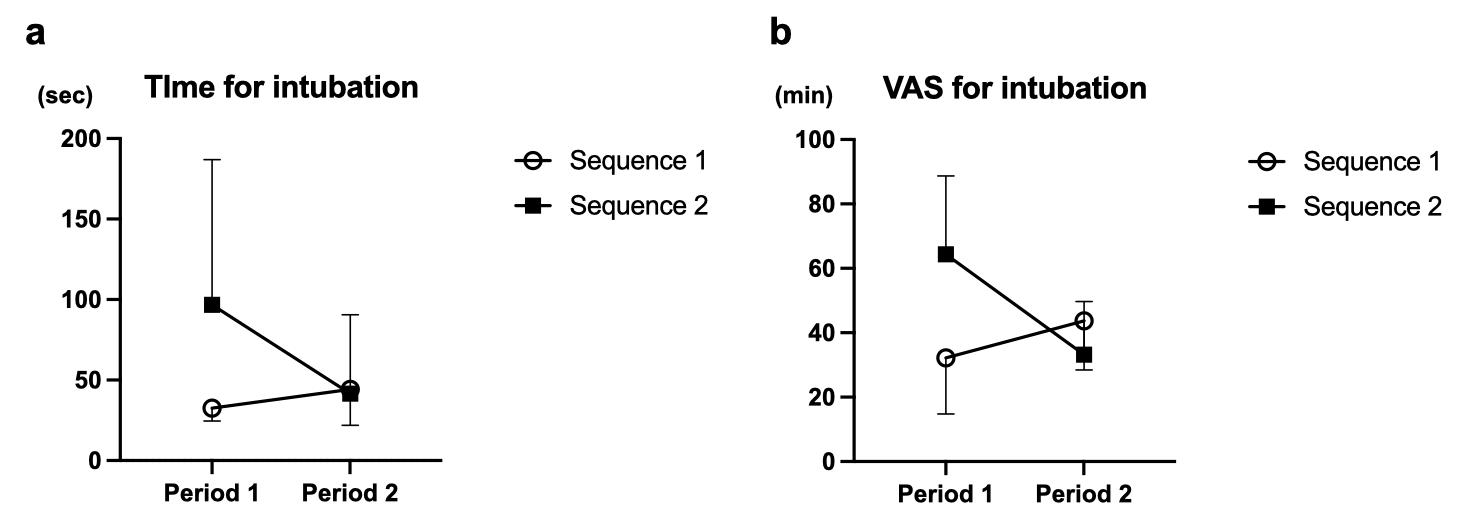


**Supplementary Fig. 1** Two-way analysis of variance of time required for fiberoptic tracheal intubation and visual analog scale for difficulty in fiberoptic tracheal intubation. a) Time needed for fiberoptic tracheal intubation. There was a significant interaction between period and Sequence (*P* = 0.011). b) Visual analog scale for difficulty in fiberoptic tracheal intubation. There was a significant interaction between period and Sequence (P < 0.001). These results mean that possible carryover effect exist in the data of time for fiberoptic tracheal intubation and visual analog scale for fiberoptic tracheal intubation.
